# A Polinton-like Virus of *C. parva* Inhibits the Population Growth of a Newly Isolated Relative of *Tethysvirus ontarioense*

**DOI:** 10.3390/v18020196

**Published:** 2026-02-01

**Authors:** George R. Thomas, Ichiro Inamoto, Christine N. Palermo, Gurshan Bajaj, Steven M. Short

**Affiliations:** Department of Biology, University of Toronto Mississauga, Mississauga, ON L5L 1C6, Canada; georgeraymond.thomas@mail.utoronto.ca (G.R.T.); ichiro.inamoto@utoronto.ca (I.I.); christine.palermo@mail.utoronto.ca (C.N.P.);

**Keywords:** algal virus, virophage, haptophyte, *Nucleocytoviricota*, *Preplasmiviricota*, *Mesomimiviridae*, *Phycodnaviridae*, CpV, CpV-PLV Moe

## Abstract

The previous discovery of genomes of Polinton-like viruses (PLVs) associated with viruses of *Chrysochromulina parva* stimulated this research to determine the biological nature of these putative viral hyperparasites. Purification of *C. parva* viruses to enable co-infection experiments led to the discovery of a previously unknown virus, CpV-BQ3, which, based on sequence information and electron microscopy, is a species of *Tethysvirus*, a genus within the *Megaviricetes*. Purification and TEM imaging of CpV-PLV Moe revealed naked icosahedral particles morphologically similar to other cultivated virophages and PLVs. Mixed-infection experiments with the putative Polinton-like virus CpV-PLV Moe demonstrated that CpV-BQ3 supports its replication, whereas the putative Phycodnavirus CpV-BQ1 does not. Further, experimental infections with differing proportions of the Moe and its helper virus CpV-BQ3 revealed a dose-effect whereby high levels of Moe had a greater negative impact on BQ3 replication compared to lower levels. Conversely, high levels of Moe relative to BQ3 provided greater protection for *C. parva,* allowing enhanced cell survival, whereas low doses of Moe did not prevent cell lysis. Overall, the results of this study demonstrated the intimate relationship of CpV-PLV Moe with the newly discovered virus, CpV-BQ3, and *C. parva*, and illustrate the complex ecology of algal viruses.

## 1. Introduction

The discovery of Sputnik [1], a satellite virus that coinfects the amoeba *Acanthamoeba castellanii* with a giant dsDNA virus, Acanthamoeba castellanii mamavirus Hal-V (*Mimivirus bradfordmassiliense*), marked the beginning of research on virophage biology and their relationship to other viruses and mobile genetic elements (MGEs). The observation that Sputnik depended on mamavirus as a helper virus while also compromising mamavirus’s replication led the researchers to introduce the term virophage to highlight its hyperparasitic lifestyle and allegorically relate it to bacteriophage [1]. Soon after, Mavirus, a relative of Sputnik that coinfects the marine phagotrophic flagellate *Cafeteria* sp. with its helper virus, CroV, was described as a virophage with evolutionary connections to MGEs in the Polinton, or Maverick, family of large DNA transposons of eukaryotes [2]. Around the same time, virophage genomes were reported from Organic Lake Antarctica metagenomes [3], and numerous examples of complete or near-complete virophage and Polinton-like virus (PLV) genomes have now been observed in metagenomes collected from around the world [4,5,6,7,8]. The isolation of Zamilon (*Sputnikvirus zamilonense*) provided evidence for the variable replication strategies of virophages because it did not appear to have a significant impact on the replication of its helper virus [9]. Together, these and the related literature triggered debate about the nature and classification of virophages, e.g., [10,11,12,13], and shared evolutionary history, e.g., [14,15,16].

Virophages and PLVs are currently classified in the phylum *Preplasmiviricota* in the kingdom *Bamfordvirae* along with dsDNA viruses of eukaryotes in the phylum Nucleocytoviricota, as defined by the International Committee on Taxonomy of Viruses; https://ictv.global/taxonomy [17]. Given the rapid pace of discovery of virophages, it is not surprising that their taxonomy has evolved since their discovery [13]. Even the use of the term ‘virophage’ has varied in the literature. It has recently been argued that the term virophage can be used in an informal sense to describe dsDNA viruses that engage in a hyperparasitic replication strategy by parasitizing dsDNA viruses of the phylum *Nucleocytoviricota*, yet the term can also be used in a formal sense to refer to viruses in the class *Virophaviricetes* in the phylum *Preplasmivircota* [18]. Related to the virophages (sensu stricto) are the Polinton-like viruses (PLVs), which are related to Polintons and encode the hallmark double-jelly-roll major capsid proteins (MCP) of dsDNA viruses in the *Nucleocytoviricota* [14,19].

Genomic analysis of a virus of the marine haptophyte *Phaeocystis globosa* (PgV-16T) led to the assembly of its ~460 kbp genome as well as a ~20 kbp virophage-like linear genome labelled PgVV [20]. Subsequent research demonstrated that PgVV was distinct from characterized Polintons and virophages, and searches for PgVV-like MCP genes in genomic and metagenomics sequences led to the identification of a novel group of PLVs [5]. Like the discovery of virophages in metagenomes from around the world, subsequent studies of PLV diversity in environmental samples and metagenomes led to the identification of hundreds of distinct PLVs in metagenomes and eukaryotic genomes [21]. Additionally, endogenous viral elements, or EVEs, related to virophages and PLVs have been identified in diverse protist genomes spanning the breadth of eukaryote clades [22], and PLVs have even been observed recently as MGEs integrated into a broad range of stony coral (order Scleractinia) genomes [23]. Recognizing the distinct identity of PLVs, the phylum *Preplasmiviricota* has been reorganized to include the established classes *Polintoviricetes* and *Virophaviricetes*, as well as two new classes *Pharingeaviricetes* and *Aquintoviricetes*, the class within which the algal-infecting PLV PgVV is placed [18]. Although viruses of the haptophyte alga *Phaeocystis globosa* (PgVs) were isolated before mimiviruses, some of these algal viruses are now considered members of the family *Mesomimividae*, and PgV-16T has been named *Tethysvirus hollandense* [24,25]. PgVV has not yet been isolated, but a recent report described the isolation and life history of another closely related PLV that coinfects *P. globosa* along with PgV-14T, a strain of *T. hollandense*. This PLV, named Gezel-14T, has been confirmed as a bona fide virus with a virophage-like lifestyle and is considered a member of *Aquintoviricetes*. Although it has a fitness impact on its helper virus, it does not appear to protect the host cell against infection [26].

A strikingly similar system of viruses and PLVs has been observed in association with the freshwater haptophyte *Chrysochromulina parva* CCMP291 [27,28,29]; it should be noted that recent studies suggest that *C. parva* CCMP291 should be reclassified as *C.* *tobinii* [30,31]. Like its relative *P. globosa*, *C. parva* is infected by viruses in the orders *Algavirales* and *Imitervirales* and is also associated with PLVs. When originally described, viruses infecting *C. parva* CCMP291 were named Chrysochromulina parva virus BQ1 (CpV-BQ1) and were putatively classified as phycodnaviruses [29]. Shortly thereafter, high-throughput sequencing resulted in assembly of a ~430 kbp virus genome encoding a PolB gene distinct from BQ1, so this virus was labelled CpV-BQ2 [28]. CpV-BQ2 is currently classified as *Tethysvirus ontarioense*, a mesomimivirus whose closest relatives are Group I PgVs [24]. Like *P. globosa*, *C. parva* may also be subject to tripartite or hyperparasitic infections because ~23 kbp genome sequences of three PLVs, CpV-PLV Larry, Curly, and Moe, were also identified during assembly of the BQ2 genome. The striking similarities of the infections of *C. parva* and *P. globosa* allow speculation that this tripartite infection system is derived from a common ancestor of these algae and predates their transition to freshwater environments. Whatever their evolutionary history, the life history and impacts on host survival and viral replication of *C. parva* PLVs have not been described. Here, we show that CpV-PLV Moe is a genuine virus with a virophage-like replication strategy that depends on co-infections with a newly discovered member of the genus *Tethysvirus*, which we have called CpV-BQ3.

## 2. Materials and Methods

### 2.1. Cell and Virus Cultivation

Non-axenic cultures of *Chrysochromulina parva* CCMP291 were originally obtained from the National Center for Marine Algae and Microbiota (NCMA; https://ncma.bigelow.org/, accessed on 6 January 2026) and have been maintained in the laboratory for over a decade following NCMA’s culturing recommendations. Briefly, naïve cells that have not been exposed to any lytic agents were maintained in 100 to 150 mL batch cultures grown in DY-V medium [32] at a temperature of 15 °C with a 12:12 h light/dark cycle at a photon flux density of 23 μmol m^−2^ s^−1^. This growth condition was also used for all infection experiments. When cells reached a maximum density of approximately 3.0 × 10^6^ cells mL^−1^, after roughly three weeks of growth, they were diluted approximately 100-fold by aseptically transferring a small aliquot of the culture into fresh medium.

Viral lysates have been maintained in the lab for over a decade since they were first reported [29]. Following the addition of 0.5 to 1 mL of undefined, filtered lysates into a mid-log phase *C. parva* culture, viruses were filter-purified after cell lysis as characterized by a loss of pigmentation in the culture, or when cell concentrations decreased to less than 3 × 10^5^ cells mL^−1^. Filter purification involved sequentially vacuum filtering lysates through 47 mm diameter, 0.50 μm nominal pore-size borosilicate glass microfiber filters (GC50, Advantec MFS, Inc. Dublin, CA, USA), and 47 mm diameter 0.45 μm pore-size PVDF Durapore^®^ membranes (MilliporeSigma, Burlington, MA, USA). Additionally, to further purify some virus preparations, lysate solutions were also filtered through a 0.1 μm pore-size Minisart^®^ (Sartorius, Shinagawa City, Tokyo) or 0.20 μm pore-size Filtropur S (Sarstedt, Nümbrecht, Germany) sterile syringe filters. Following filtration, lysate stocks were stored in the dark at 4 °C for up to 7 months.

### 2.2. Virus Isolation and Cultivation

To isolate individual virus types from mixed lysates, a combination of serial propagation, end-point dilution, and differential filtration was used, and the abundances of known viruses (i.e., CpV-BQ1 and -BQ2, and CpV-PLV Larry, Curly, and Moe) were monitored in all lysate samples using qPCR (described below). Every lysis experiment conducted during virus isolation efforts was initiated from a single culture stock, which was split into two: one serving as the no-infection control and the other infected with putative lytic agents. Starting with a mixed viral stock that included all known viruses, viruses were serially propagated in two parallel lineages by adding 0.5 mL of 0.45 or 0.20 μm pore-size syringe-filtered lysates into mid-log phase *C. parva* cultures and then filtering and transferring the resulting lysates into fresh mid-log phase cultures. After five generations, lysates from each lineage were subjected to further purification via endpoint dilution in 96-well microtiter plates. The entire 300 μL sample from wells at the highest dilution level, which cleared, was then inoculated into 100 mL cultures to test their lytic activity, and was subsequently propagated again for several generations. Finally, a portion of these lysates obtained after end-point dilution was filtered through 0.1 μm pore-size syringe filters to determine if this filtration step eliminated lytic activity.

For the sake of brevity, the viruses CpV-BQ1, -BQ2, and the newly discovered CpV-BQ3 will henceforth be referred to as simply BQ1, BQ2, and BQ3. Likewise, the PLVs CpV-PLV Larry, CpV-PLV Curly, and CpV-PLV Moe will be referred to as Larry, Curly, and Moe.

### 2.3. Sequence Analysis

An infectious *C. parva* lysate containing no detectable levels of BQ1 or BQ2, or Larry, Curly, or Moe was concentrated by centrifugation at 31,000 rpm for 1 h at 20 °C using an SW32Ti rotor (Beckman-Coulter, Mississauga, ON, Canada). Pelleted material was resuspended in 10 mM Tris-Cl, pH 8.5, and DNA was extracted from 300 µL of the resuspended material using a Promega Maxwell RSC Viral Total Nucleic Acid extraction kit. Final DNA concentrations were measured using a Qubit dsDNA HS assay kit (Thermo Fisher Scientific, Waltham, MA, USA). Sample library preparation was performed using an Illumina DNA Prep kit and UDI indices, and libraries were sequenced from paired ends (2 × 151 bp) on a NextSeq 2000 (Illumina, San Diego, California, USA.) by SeqCenter (Pittsburgh, PA, USA). Demultiplexing, quality control, and adapter removal were performed by SeqCenter using BCL-convert version 3.9.3 (Illumina, San Diego, California, USA.).

Additional quality control was performed using Sickle version 1.33 [33] with a quality threshold value of 30 and a read length cut-off value of 50. Reads were assembled using default parameters in metaSPAdes version 3.15.5 (https://www.ncbi.nlm.nih.gov/pmc/articles/PMC5411777/, accessed on 6 January 2026). BLASTx version 2.13.0. searches against the October 2022 NCBI-nr database (https://ftp.ncbi.nlm.nih.gov/blast/db/FASTA/nr.gz, accessed on 31 October 2022) were performed using DIAMOND version 2.0.15 [34] with frameshift alignment and more sensitive modes activated. MEGAN6-LR version 6.24.1 [35] was used to annotate contigs using the February 2022 protein accession mapping file (https://software-ab.informatik.uni-tuebingen.de/download/megan6/welcome.html, accessed on 31 January 2026) with long-read mode activated and bit score and E value cutoffs of 100 and 10^−6^, respectively. The inferred amino acid sequences of annotated genes from Illumina sequencing were compared to GenBank NR using BLASTp. Homologous sequences of common *Nucleocytoviricota* marker genes encoding PolB (DNA polymerase family B), A32 (packaging ATPase), and VLTF3 (Poxvirus Late Transcription Factor) proteins, e.g., [25], were analyzed phylogenetically using MEGA version 12 [36]. For all phylogenetic analyses, the percentage of replicate trees in which the associated taxa clustered together (500 replicates) is shown next to the branches [37]. The initial trees for the heuristic searches were selected by choosing the trees with the superior log-likelihood between Neighbor-Joining [38] and Maximum Parsimony trees. The NJ trees were generated using a matrix of pairwise distances computed using the Jones–Taylor–Thornton model [39], whereas the MP trees had the shortest lengths among 10 MP tree searches, each performed with a randomly generated starting tree.

### 2.4. Transmission Electron Microscopy

Samples containing no detectable viruses yet retaining lytic activity, or samples containing high concentrations of Moe, as inferred from qPCR, were concentrated by centrifugation at 33,000 RPM for 1 h for samples of an unknown lytic agent, or 11,000 RPM for 1 h for Moe using the SW32Ti Rotor in an Optima L-80XP Ultracentrifuge (Beckman-Coulter, Mississauga, ON, Canada). Pelleted material was resuspended in 10 mM Tris-Cl, pH 8.5. Carbon-coated copper grids (FCF400-Cu-UB, Electron Microscopy Sciences, Hatfield, PA, USA) were glow-discharged immediately before use, and 10 µL of concentrated samples was applied to the grid and allowed to sit for 10 min. The grid was then washed with double-distilled H_2_O three times, followed by staining using 0.5 to 2% uranyl acetate for 30 s. Stained grids were visualized using a Talos L120C transmission electron microscope (Thermo Fisher Scientific) at the Microscopy Imaging Laboratory, Temerty Faculty of Medicine, University of Toronto.

### 2.5. Cell Counts, MPN, and qPCR

Cell counts were performed on culture samples by adding 20 μL of Lugol’s iodine to 200 μL samples immediately after collection. Samples were stored in the dark at room temperature and counted within 2 weeks following collection using a Bright-Line hemocytometer (Hausser Scientific, Horsham, PA, USA) and a ZEISS Primostar 3 microscope equipped with an Axiocam 105 digital camera (Carl Zeiss Canada Ltd., Toronto, ON, Canada). Images of five of the nine grid blocks of the hemocytometer were captured, and the images were processed in Fiji [40], where a pseudo–flat field correction (blurring parameter = 50) was applied to even out background illumination, followed by thresholding to highlight cells for counts.

The infectivity of stocks of the viruses BQ3 or BQ1 was estimated using the Most Probable Number (MPN) assay. Eleven 10-fold serial dilutions of BQ3 or BQ1 lysates were prepared in sterile DY-V medium, and 50 μL of each virus dilution was inoculated into 16 wells of a 96-well microtiter plate containing 250 μL of mid-log phase *C. parva* culture. In each plate, two columns of wells with 50 μL of sterile DY-V media added served as no-infection controls. All MPN plates were incubated in the same growth conditions used for all *C. parva* cultures. Plates were checked and scored for lysis (i.e., well clearing) regularly up to 21 days to ensure that lysis patterns had stabilized. The number of wells lysed at each dilution level was used to estimate the infectivity of virus stocks of BQ3 or BQ1 with the Excel-based MPN calculator, MPN_ver6 [41]. Because infection with Moe alone did not lyse *C. parva* cultures, infectivity estimation via MPN was not possible. Therefore, the abundance of Moe was only estimated using qPCR.

Gene copy abundances were used to infer virion abundance using the 5′ nuclease assay targeting PolB genes for BQ1, BQ2, and BQ3, and the major capsid protein (MCP) genes for Larry, Curly, and Moe (Table 1). Quantitative PCR (qPCR) was conducted as previously described for BQ1 and other freshwater viruses [29,42]. Briefly, 25 μL reactions contained 1 X PCR buffer [20 mM Tris-HCl (pH 8.4) and 50 mM KCl], 5.0 mM MgCl_2_, 200 μM each dNTP, 400 nM each forward and reverse primer, 200 nM of TaqMan^®^ probe, 0.625 U Invitrogen Platinum^®^ Taq DNA polymerase (Thermo Fisher Scientific), 1 X ROX Reference Dye (Life Technologies, Thermo Fisher Scientific, Waltham, MA, USA), and 2 μL of virus sample. Virus samples were used directly in the qPCR reaction after storage at −20 °C for at least 15 h. Reactions were cycled in a Stratagene MX3000P thermal cycler (Agilent Technologies Canada Inc., Mississauga, ON, Canada) with the following parameters: 95 °C for 5 min, and 40 cycles of 95 °C for 15 s followed by 60 °C for 1 min. Primer and probe sets not previously reported (Table 1) were designed and validated as previously described [42,43].

### 2.6. Infection Experiments

Experimental treatments were distributed across four independent trials due to logistical reasons related to growth chamber capacity and to minimize handling time for sample collection. Trials were set up with experimental treatments established in triplicate using disposable 75 cm^2^, 250 mL capacity, vented, Polystyrene cell culture flasks (Sarstedt). For each experiment, the starting abundance of *C. parva* cells was approximately 3.5 × 10^5^ cells mL^−1^. Each treatment involved inoculating no more than 1 mL stocks of CpVs and/or Moe into 100 mL of *C. parva* cultures. Each experimental trial included a no-infection control, as well as a lytic infection control with BQ3 inoculated at an MOI of 0.01 infectious units per cell; the same MOI was used for all mixed infections with BQ3 or BQ1. Experimental treatments included co-inoculation of *C. parva* with BQ3 and Moe at defined ratios, as determined via qPCR. The experimental treatments included across trials were as follows: (1) BQ3 + Moe 1:1000 and Moe alone; (2) BQ3 + Moe 1:900 and BQ3 + Moe 1:10; (3) BQ3 + Moe 1:500 and BQ1 + Moe 1:500; (4) BQ3 + Moe 1:200. The concentration of the Moe inoculum used in the ‘Moe alone’ treatment was the same gene copy concentration of the Moe inoculum in the 1:1000 co-infection treatment. The BQ1 inoculum was matched to the gene copy number of the BQ3 inoculum used in its respective infection control.

Culture flasks were arranged within the growth chamber to maximize variability of irradiance for each set of replicates. Sampling was performed consistently at the same time each day when two 200 μL samples were aseptically collected from each flask for cell counts and qPCR. Samples collected for cell counts were enumerated within 2 weeks following collection, whereas samples collected for qPCR were stored at −20 °C immediately upon collection and until further analysis; qPCR analysis was conducted within 3 weeks of sample collection.

### 2.7. Statistical Analyses

For each experimental run, *C. parva* cell abundances on day 9 were compared among treatments using Welch’s ANOVA, followed by Games–Howell post hoc testing. BQ3 replication was analyzed similarly, but was based on comparisons of BQ3 abundances in co-infections relative to the BQ3-only infection control in the corresponding trial. Data were power-transformed prior to analysis to improve normality, and the transformed residuals were assumed to follow an approximately normal distribution based on Q–Q plots. All post hoc tests were performed using the rstatix package in R. To evaluate whether Moe affected BQ3 replication, log_10_-transformed BQ3 abundances from day 9 samples were compared between the BQ3-only infection controls and corresponding BQ3 + Moe co-infections using a Welch’s two-sample *t*-test. A normal distribution of the log-transformed data was assumed. To conservatively account for multiple comparisons, a Bonferroni-adjusted significance threshold of α = 0.025 was applied to all tests.

## 3. Results

### 3.1. Quantitative PCR Assay Validation

Primer and probe sets used in this study were validated in amplifications with cloned gene fragments added to the reaction at a concentration of 5.0 × 10^9^ gene copies mL^−1^ (Table 2). Considering the Cq value as an indication of amplifiability of different template molecules, the primer and probe set targeting Curly was the most prone to off-target amplification, producing a Cq value of 20.3 when amplifying Moe’s MCP gene compared to a Cq of 15.0 for its intended target template. The primer and probe set targeting Moe was the most specific, producing no amplification for any target except Curly; in this case, the on-target Cq was 17.2, whereas the off-target Cq was 36.0. Similarly, the primer and probe set targeting the newly discovered BQ3 virus (see below) produced no amplification for any templates except BQ2 and Moe. For this primer and probe set, the on-target Cq was 17.2, whereas the off-target Cq values were 27.0 and 36.8 for the templates of BQ2 and Moe, respectively.

### 3.2. Virus Isolation

To isolate the individual *Chrysochromulina parva* viruses, a virus stock containing a mixture of all known viruses and PLVs (i.e., BQ1, BQ2, Larry, Curly, and Moe), as confirmed via qPCR, was propagated through multiple serial infections of *C. parva* culture. We hypothesized that fitness differences between these viruses when grown in specific laboratory conditions would result in the enrichment of certain viruses after several generations of infection. After 5 generations of infection in both parallel propagation lineages (i.e., 0.45 vs. 0.20 μm pore-size filtration), all viruses except for Moe became undetectable by quantitative PCR, yet filtered medium from each series still lysed *C. parva* cultures. A portion of the lytic sample collected from the 0.20 μm pore-size filtration was further filtered through a 0.10 μm pore-size filter. The 0.10 μm filtrate contained Moe at high abundance (Cq = 19.5), yet it did not cause culture lysis. On the other hand, when the 0.10 μm filter itself was soaked in sterile DY-V medium overnight, and a 1/10 *v*/*v* of this was added into a mid-log phase culture, *C. parva* lysis was observed. None of the known viruses was detected in this culture lysate.

We then used ‘dilution to extinction’ (via MPN) to further purify and isolate lytic agents present in the fifth-generation lysates from each of the parallel propagation lineages. From each MPN assay, a sample at the highest dilution level that produced visible cell lysis was selected for further analysis and propagation. These two samples were propagated through an additional four generations of propagation following the same filtration scheme. Of all known viruses, only Moe was detectable in the lysates derived from the 0.20 μm filtration series, whereas none of the known viruses (i.e., BQ1, BQ2, Larry, Curly, or Moe) were detectable in the 0.45 μm filtration series. Nucleic acids were extracted from a portion of the filtered lysate from the 0.45 μm filtration series and were sent for Illumina sequencing.

### 3.3. Sequence Analysis and Phylogeny of an Unknown Lytic Agent

Sequence analysis of nucleic acids recovered from the last generation of lysate from the 0.45 μm filtration series is ongoing. Preliminary analysis resulted in the assembly of four large (i.e., > 50 kbp) contiguous sequences, which, via BLAST analysis, were most closely related to the published genome of BQ2 [28] and represent the genome sequences of a newly discovered *C. parva*-infecting virus we have called CpV-BQ3. Because these contigs cannot yet be assembled into a single virus genome, they are not reported here. However, several hallmark virus sequences, including those encoding PolB (DNA polymerase family B), A32 (packaging ATPase), and VLTF-3 (Poxvirus Late Transcription Factor) proteins, were identified in two of the largest contigs. These gene sequences have been submitted to GenBank and have been assigned the accession numbers PX584302 (PolB), PX584303 (A32), and PX584304 (VLTF3). BLAST comparisons of these sequences to sequences in GenBank demonstrated that all three sequences are nearly identical to sequences of the *C. parva*-infecting virus BQ2, or *Tethysvirus ontarioense*. The DNA sequence of BQ3’s PolB gene is 93% identical over 3865 bp to the BQ2 PolB gene, its A32 gene is 95% identical over 839 bp, and its VLTF3 gene is 93% identical over 1148 bp.

**Figure 1:**
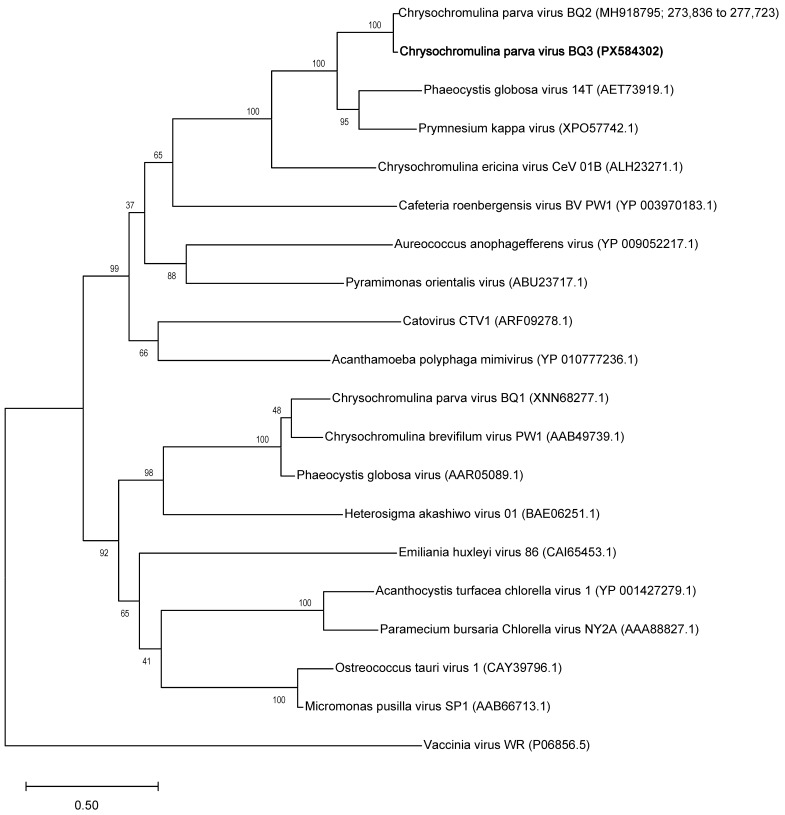
Phylogenetic analysis of virus PolB genes. The phylogeny was inferred using the Maximum Likelihood method and Jones–Taylor–Thornton model of amino acid substitutions [44], and the tree with the highest log likelihood (−44,597) is shown. The final dataset for analysis included 20 amino acid sequences with 2380 positions.

Phylogenetic analyses of these gene sequences corroborate earlier analyses of members of the *Nucleocytoviricota* like *Tethysvirus ontarioense* (BQ2), reinforcing the monophyly of haptophyte (i.e., prymnesiophyte) infecting viruses within the *Mesomimiviridae*. The PolB genes of BQ3 and BQ2 were placed as sister taxa in a clade most closely related to *Phaeocystis globosa* virus 14T and *Prymnesium kappa* virus, and the *Chrysochromulina ericinia* virus CeV-01B, or *Tethysvirus raunefjordense* (Figure 1). This same close relationship of BQ3 sequences to genes from other marine haptophyte-infecting viruses is echoed in the phylogenies of the A32 and VLTF-3 genes (Figures S1 and S2, respectively).

### 3.4. TEM

**Figure 2:**
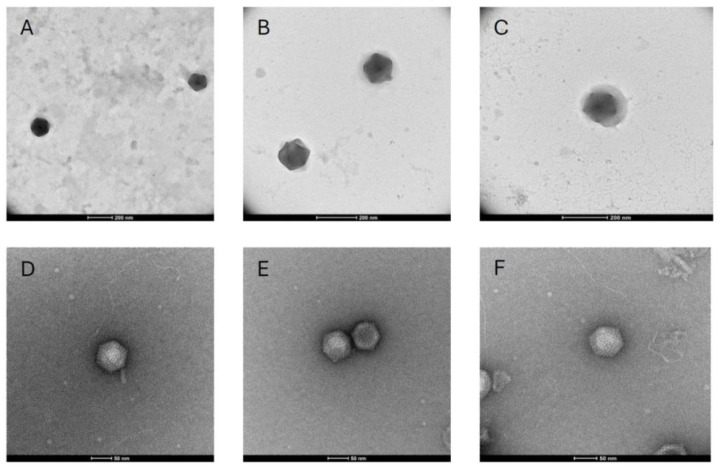
Transmission electron microscopy of viral lysates with unmodified scale bars included at the bottom of each image. (**A**–**C**) Micrographs obtained from samples with highly abundant BQ3 viewed at 36,000× magnification (**A**) or 57,000× magnification (**B**,**C**). (**D**–**F**) Micrographs obtained from samples with highly abundant Moe viewed at 120,000× magnification.

Throughout the process of virus isolation, filtered lysate samples were examined using transmission electron microscopy. Examination of the final lysate obtained from the 0.45 μm filtration series, from which a portion was used for DNA extraction and sequencing, revealed numerous particles with roughly the same icosahedral shape and diameter. The largest particles observed in these grids were approximately 150 nm in diameter (Figure 2A,B), and the smallest particles were approximately 140 nm in diameter (Figure 2C). Some particles appeared to be surrounded by an amorphous outer layer of material associated with a more rigid appearing core that is similar in size to particles lacking this layer (Figure 2C). Particles with appearances like these ~150 nm particles (and with both naked and sheathed appearance) were also observed in preparations from earlier stages of isolation, and retrospectively, all these samples contained highly abundant BQ3 PolB genes, as inferred by qPCR. On the other hand, TEM visualization of samples from the 0.20 μm filtration series, for which only Moe was detectable by qPCR (prior to the discovery and sequencing of BQ3), was dominated by negatively stained, icosahedral particles approximately 70 nm in diameter (Figure 2D–F). All these smaller particles appeared to be rigid naked particles, with no outer material associated with them.

### 3.5. Growth Experiments

**Figure 3:**
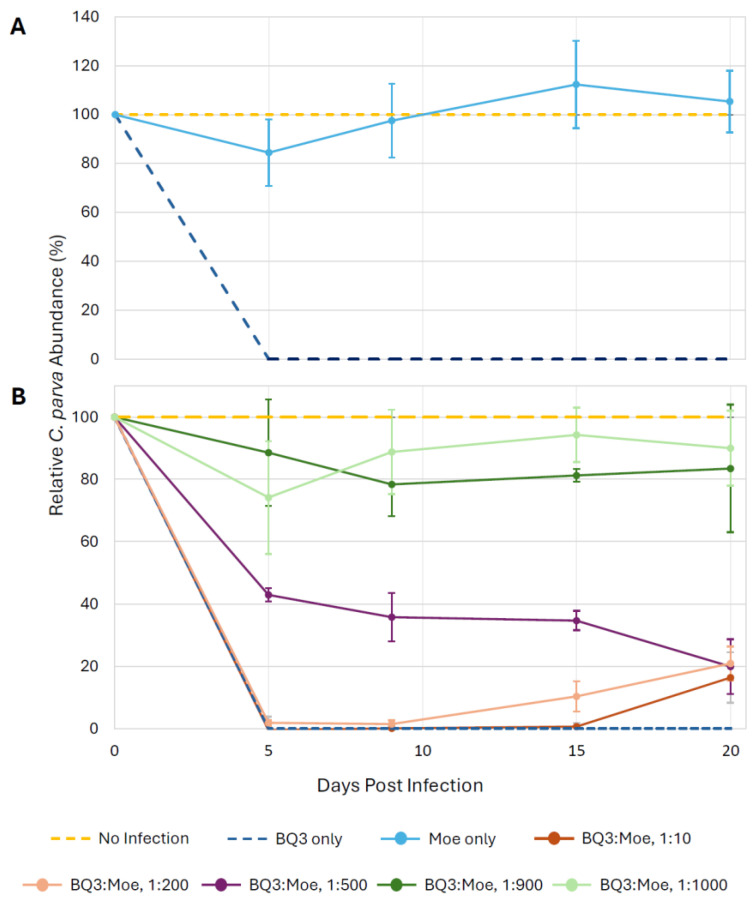
Relative growth of C. parva cultures over 20 days. Cell growth was expressed as a percentage of the mean growth observed in triplicate no-infection control flasks; cell counts were normalized across 4 experimental trials utilizing the equation: (Treatment-Infection Control)/(Negative Control-Infection Control) × 100%. (**A**) Relative *C. parva* growth when only Moe was inoculated into triplicate flasks. (**B**) *C. parva* growth when Moe was inoculated at different ratios relative to BQ3. In both panels, error bars represent propagated standard deviations of growth estimates from triplicate flasks, incorporating variability from respective no-infection and BQ3-only control replicates.

To facilitate visual comparisons of data from all growth experiment trials, the relative abundance of *C. parva* cells was plotted by expressing the abundance of cells in experimental treatments as a proportion of the abundance of cells in the no-infection controls in the same trial (Figure 3). In every experimental trial, the infection with BQ3 alone caused complete lysis of *C. parva* cultures within 5 days post-infection. However, infections with Moe alone did not appear to cause lysis as cell abundances remained close to the no-infection controls over 20 days (Figure 3A). However, depending on the proportion of Moe in the mixed infection treatments, relative cell abundances differed from the BQ3 infections. Relative cell abundances in the experimental treatments with the highest proportions of Moe (1:1000 and 1:900 BQ3/Moe) remained high throughout the experiment, with final abundances above 80% of the abundance of the growth controls (Figure 3B). Cells treated with BQ3/Moe added at 1:500 appeared to experience some lysis, with abundances dropping to just over 40% by the fifth day of the experiment and ending at around 20%. Relative cell abundances in treatments with the lowest proportions of Moe (1:10 and 1:200 BQ3/Moe) were similar to infections with BQ3 alone, except cell growth appeared to recover slightly by the end of the experiment, with abundances reaching approximately 20% of the growth control on day 20 (Figure 3B).

Statistical analyses of cell growth were performed on the raw abundance data (Figure S3) on the 9th day of each experimental trial. Welch’s ANOVA detected significant differences in day-9 cell abundances among treatments within each experimental trial, and Games–Howell post-hoc tests revealed clear pairwise patterns. In the first experimental trial, *C. parva* cell abundances in the BQ3/Moe 1:1000 mixed infection and Moe-only treatment were significantly higher than in BQ3-only infections (*p* < 0.001) and did not differ from no-infection controls (*p* = 0.14). In the second trial, cell abundances in the BQ3/Moe 1:900 treatment were significantly higher than BQ3-only infections (*p* < 0.001) and comparable to no-infection controls (*p* = 0.13), whereas abundances in the BQ3/Moe 1:10 treatment differed from no-infection controls (*p* < 0.001) but not from BQ3-only infections (*p* = 1.0). In the third trial, cell abundances in the BQ3/Moe 1:500 treatment differed significantly from both no-infection controls (*p* = 0.026) and BQ3-only infections (*p* = 0.001). In the final trial, cell abundances in the BQ3/Moe 1:200 treatment differed from no-infection controls (*p* < 0.001) but not from BQ3-only infections (*p* = 0.23).

**Figure 4:**
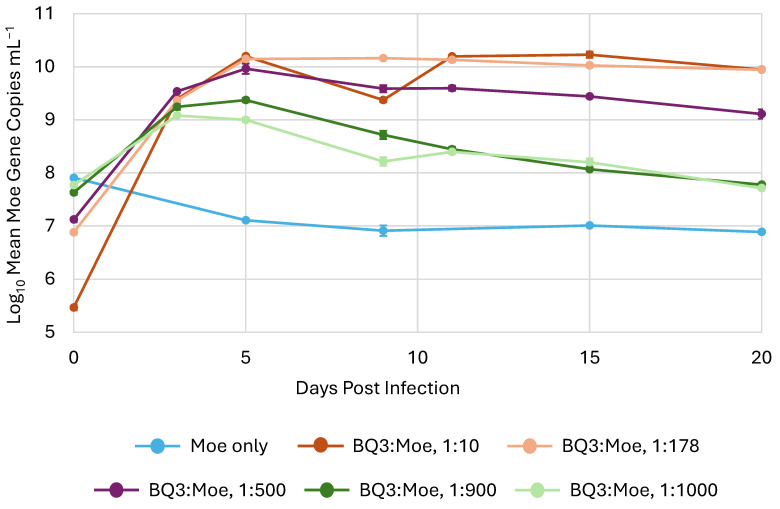
The replication of Moe in mixed infections. Moe’s replication varied in triplicate experimental flasks inoculated with different ratios of Moe relative to BQ3, which was held at a constant multiplicity of infection. The legend shows the approximate ratio of BQ3/Moe for each treatment based on qPCR estimates. The error bars correspond to the standard deviation of Moe gene copies estimated in triplicate flasks, and where they are not visible, they are smaller than the data marker.

In all experimental trials, the abundance of Moe, inferred from qPCR, increased after the initial infection (i.e., day 0) in all mixed infection treatments with Moe and BQ3 (Figure 4). However, after an initial approximately 10-fold increase, the abundance of Moe gene copies decreased back to the original starting abundance in both the 1:900 and 1:100 BQ3/Moe mixed infections. On the other hand, Moe gene abundances in the 1:10, 1:200, and 1:500 mixed infections increased by approximately 4.5, 3, and 2 orders of magnitude, respectively, after the 20-day incubation. Finally, the abundance of Moe MCP gene copies decreased from approximately 10^8^ copies mL^−1^ to approximately 1 × 10^7^ copies mL^−1^ in the replicate flasks with Moe alone.

**Figure 5:**
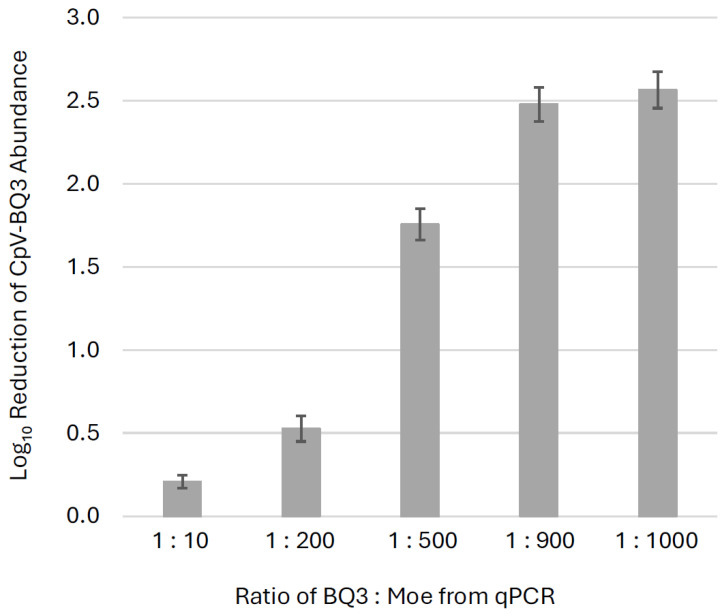
Inhibition of BQ3 replication. The fold-change reductions of BQ3 abundances in mixed infections relative to corresponding BQ3-only infections are shown for different ratios of BQ3/Moe after 9 days post-inoculation. Estimates represent the mean fold-reduction of BQ3 abundance in triplicate flasks relative to the mean in triplicate BQ3 infection controls. The error bars correspond to the standard deviation of the estimates, and ratios of BQ3/Moe were based on qPCR estimates of virus abundances.

Across all trials, the presence of Moe significantly reduced BQ3 replication by day 9, with BQ3-only infections yielding higher BQ3 gene copy abundances than all corresponding mixed-infection treatments (*p* ≤ 0.002). To visualize this effect, fold-change reductions in BQ3 gene copies relative to BQ3-only infections were plotted (Figure 5). BQ3 replication was reduced by ~300-fold in treatments at the highest proportions of Moe (BQ3/Moe 1:1000 and 1:900), ~50-fold at 1:500, ~3-fold at 1:200, and ~2-fold at 1:10. Welch’s ANOVA demonstrated a significant difference in BQ3 reduction across treatments. A Games–Howell post-hoc test demonstrated that the only non-significant comparison was between the 1:900 and 1:1000 BQ3/Moe treatments (*p* = 0.637), indicating that these two mixtures resulted in similar levels of BQ3 reduction. All other pairwise comparisons were statistically significant, with BQ3 replication significantly reduced in the 1:1000, 1:900, 1:500, and 1:200 treatments relative to the 1:10 treatment (*p* < 0.01 in all cases).

**Figure 6:**
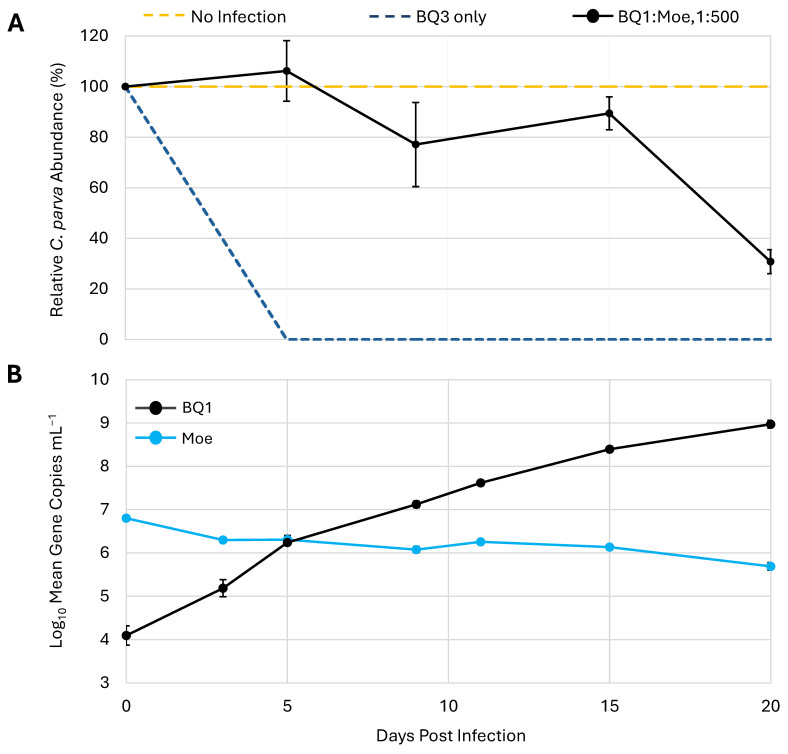
Replication of Moe in the presence of the virus BQ1. (**A**) *C. parva* growth is shown as a percentage relative to no-infection control flasks. Inoculation with BQ3 alone was used as an infection control. (**B**) The abundances of BQ1 and Moe were estimated using qPCR and are shown for triplicate flasks inoculated with both BQ1 and Moe at a ratio of 1:500, BQ1/Moe. The error bars represent the standard deviation of triplicate flasks, and where they are not visible, they were smaller than the data marker.

To determine if the effect of Moe on the replication of the putative Phycodnavirus BQ1 was similar to its effects on the Mesomimivirus BQ3, a mixed infection with a 1:500 mixture of BQ1 and Moe was included in the third trial. This coinfection resulted in a different lysis pattern compared to the infections with a 1:500 mixture of BQ3/Moe (Figure 3B and Figure 6A). In the mixture with BQ1, *C. parva* abundances remained close to 80% of the no-infection control until the 20th day of the experiment, when cell abundances dropped to approximately 30 ± 4% of the *C. parva* abundances in the no-infection control. The abundances of BQ1 and Moe in the BQ1: Moe mixed infection were monitored over the 20-day sampling period via qPCR (Figure 6B). BQ1 appeared to replicate throughout the sampling period of the experiment, steadily increasing from the initial inoculum of 1.3 × 10^4^ gene copies mL^−1^ up to 9.5 × 10^8^ gene copies mL^−1^ on day 20. In contrast, there was no evidence of Moe’s replication as its abundance decreased slightly from an initial inoculum of 6.4 × 10^6^ gene copies mL^−1^ to a final abundance of approximately 5.0 × 10^5^ gene copies mL^−1^.

## 4. Discussion

The motivation for this study was to determine the relationship between the algal viruses BQ1 and BQ2 and the Polinton-like viruses Larry, Curly, and Moe. To accomplish this, these viruses needed to be purified from the mixed cultures in which they had been propagated since their discovery; all originated from a single Lake Ontario, Canada water sample collected in 2011 [29]. Efforts to isolate individual viruses were facilitated by gene-specific qPCR, which allowed the abundance of each virus to be monitored in mixed infections. Quantitative PCR assay validation experiments demonstrated that all primer and probe sets performed with appropriate specificity when amplifying cloned gene fragments from purified plasmid preparations. To provide a conservative estimate of the signal generated from off-target amplifications, the concentration of plasmid templates used in validation experiments was generally higher than observed in experimental infections; only Moe MCP genes exceeded 10^9^ gene copies mL^−1^ in any experimental infection (Figure S3). The least specific primer and probe set (i.e., generating the most off-target amplification) targeted Curly. This set amplified Moe genes, albeit with approximately 40-fold reduced sensitivity based on the Cq difference of 5.3 between off-target and on-target amplification. Most importantly for the experimental infections which focused on interactions of BQ3 and BQ1 with Moe, the primers and probes for these templates were all at least 1000 times less sensitive to non-target templates (i.e., Cq difference ~ 10), and the primers and probes for Moe did not amplify any off-target genes except Curly and that with nearly a million-fold reduced sensitivity (Cq difference was ~19).

Quantitative PCR of known viruses (BQ1 and 2, and Larry, Curly, and Moe) was used to monitor changes in virus abundance through serial propagation and dilution-to-extinction experiments and provided evidence that virus stocks included a previously unknown virus. In the lineage propagated after filtration through 0.45 µm pore-size membranes, all known viruses dropped below detection. On the other hand, the lineage filtered through 0.2 µm membranes, only Moe remained detectable, maintaining relatively high abundances in the lysates (~1.0 × 10^9^ copies mL^−1^). Together, these results stimulated the hypotheses that Moe could replicate on its own, or viral lysates contained another lytic agent that had not been discovered in earlier characterizations of *C. parva* viruses [28,29]. To test these hypotheses, we sequenced nucleic acids from a lysate with no detectable viruses and conducted the mixed-infection experiments described herein.

Illumina sequencing led to the identification of three hallmark viral sequences in contigs of a previously unknown *C. parva*-infecting virus. For the sake of consistency with previous *C. parva* isolates from Lake Ontario, Canada, we have informally named this virus CpV-BQ3 because it is the third *C. parva* virus isolated from the Bay of Quinte water sample that led to the discovery of BQ1 and BQ2 [28,29]. The fact that BQ3 seems to outcompete BQ2 in serial propagation experiments suggests it has a fitness advantage over BQ2 when replicating in laboratory conditions. Presently, BQ1 has been isolated, and its genome sequence has been published [27], whereas BQ2 has not yet been isolated but remains detectable in mixed cultures of CpVs. Direct comparisons of viral fitness will be possible once BQ2 is isolated.

Maximum-likelihood phylogenies of the PolB, VLTF3, and A32 genes of BQ3 (Figure 1, Supplementary Figures S1 and S2) demonstrated their close relationship to homologous genes of *Tethysvirus ontarioense*, or BQ2. For viruses in the order *Imitervirales*, it has been suggested that two viruses should be considered the same species if pairwise average nucleotide identities (ANI) are above 95% for more than 75% of the predicted genes in each genome [24]. Given that the hallmark genes of BQ3 were 93 to 95% identical to BQ2 genes, it remains unclear if CpV should be considered a strain of *T. ontarioense* or a new species of *Tethysvirus*. Nevertheless, these phylogenies unambiguously placed genes of BQ3 as sister to BQ2 genes, and moreover, that the relationships between BQ2 and BQ3 and other haptophyte-infecting mesomimiviruses of *Phaeocystis globosa* and *Haptolina ericinia* (syn. *Chrysochromulina ericinia*) were consistent with established phylogenies, e.g., [20,24,25,29,45]. Hence, it is tenable to propose that BQ3 is a species of *Tethysvirus*, a *Varidnaviria* genus in the family *Mesomimiviridae*. Using the PolB gene sequence of BQ3, a new qPCR primer and probe set was designed and validated, providing a tool to monitor its abundance in lysates generated from mixed virus infections.

A portion of the BQ3-containing lysate that was analyzed by Illumina sequencing was imaged using transmission electron microscopy. Numerous ~150 nm in diameter, icosahedral virus-like particles were observed in these samples. Some, but not all, of the particles observed in the same grids were surrounded by an outer sheath layer (Figure 2B,C). Additionally, retrospective examination of samples using the newly designed BQ3 qPCR primers and probes demonstrated that earlier samples from this lineage also contained similarly sized particles with the same morphologies, i.e., both naked and sheathed particles were observed in lysates with highly abundant BQ3 (Supplementary Figure S4). This strongly suggests that these virus-like particles represent the BQ3 virion. It is not clear if this ‘sheath layer’ is a structural component of the virions, like the fiber layer observed in structural studies of Mimivirus [46], but the fact that this layer has been seen in different preparations of BQ3 provides evidence that it is a virion component rather than an artefact. Further experimental infections and characterization of purified BQ3 virions will be needed to resolve the structure of this unusual outer layer and determine if it is an essential component of virions.

During initial isolation attempts, an additional lineage of lysates was propagated using filtration through 0.2 µm pore-size filters. After five generations, the only known virus at the time (i.e., BQ1, BQ2, Larry, Curly, Moe) detectable was Moe. Imaging this ‘Moe-only’ sample revealed the presence of virions with icosahedral morphology ~70 nm in diameter. These particles were more easily negatively stained than BQ3 particles, and appeared as well-defined, naked particles similar in morphology and size to other *Preplasmiviricota* [18] virions like PLV Gezel-14T, which co-parasitizes *Phaeocystis globosa* along with the *Mesomimiviridae* PgV-14T [26], the Mimivirus virophages Sputnik and Zamilon, and the CroV virophage Mavirus [11]. Typically, Polinton-like viruses such as Moe require a helper virus for their replication and cannot cause cell lysis on their own. Indeed, lysate samples with highly abundant Moe gene fragments lost their ability to lyse *C. parva* cells when passed through 0.1 µm pore-size filters, and Moe remained highly abundant in these filtrates. On the other hand, material retained on the 0.1 µm filters was lytic and infectious, providing evidence for the existence of an unknown virus, and one that presumably supported replication of Moe. This stimulated reanalysis of ‘Moe-only’ samples following the discovery of BQ3 and demonstrated that these samples contained abundant BQ3 PolB genes as well. Together, these results led to the hypothesis that BQ3 was the helper virus permitting Moe’s replication and motivated the mixed infection experiments reported herein.

Before discussing the mixed-infection experiments, it is important to consider the statistical approaches used in the analyses. Logistical constraints of this study were related to the space available in growth chambers and the ability to collect samples at each timepoint from replicate flasks of each treatment while minimizing the handling of multi-day incubations. These constraints required treatments to be conducted in separate experimental trials, each including positive and negative infection controls. Since treatment replication was limited to triplicates, formal tests of normality could not be performed reliably; therefore, observed viral and cell abundances were assumed to approximate normality. Within each experimental trial, all replicates were inoculated using the same viral stock, ensuring consistency of initial conditions and supporting this assumption despite small sample sizes. For analyses employing ANOVA comparisons, residuals were normalized using power transformations (i.e., square-root or fractional power transformations), with an appropriate transformation selected to satisfy model assumptions based on model diagnostics (Q–Q plots). Variance homogeneity was not assumed for any comparisons, as mixed infections with higher ratios of Moe to BQ3 resulted in larger variances in cell and virus abundances. Accordingly, Welch’s tests were used instead of equal-variance alternatives, providing a more conservative, robust test for these comparisons. When evaluating the effect of the virophage Moe on BQ3 replication, a Bonferroni-adjusted significance threshold (α = 0.025) was applied to account for multiple comparisons; each experimental trial involved either one or two comparisons between the BQ3 infection control and the corresponding co-infection treatment.

Overall, mixed infections demonstrated that Moe’s replication depended on its *Mesomimividae* helper virus, BQ3. When Moe was added to *C. parva* cultures alone, the cultures continued to grow at abundances that did not differ from mock-infected controls (Figure 3A, Supplementary Figure S3A). In these cultures, Moe’s gene abundance dropped 10-fold over the first 5 days of the incubation and remained at this reduced abundance throughout the course of the 20-day incubation (Figure 4). The observed loss of Moe gene copies through this experiment cannot be explained at this time, but it is plausible that Moe particles and amplifiable gene fragments were destroyed during incubation by various biological and/or abiotic sources of decay known for viruses in natural environments [47]. Nevertheless, these results indicate that Moe does not replicate when added to C. parva cultures alone.

On the other hand, when Moe was added to *C. parva* cultures in the presence of BQ3, Moe abundances always increased. Preliminary experiments suggested that Moe affected both *C. parva* growth and virus replication in a density-dependent manner. Hence, we conducted a series of experimental trials, which included mixed infections with BQ3 and Moe added in proportions ranging from 1:10 to 1:1000. These proportions were created by varying the inoculum of Moe and were empirically confirmed in all replicate incubations at time zero. In cultures receiving the lowest inoculum of Moe (1:10), Moe abundances increased 50,000-fold over the 20-day incubation, whereas in cultures receiving the highest inoculum (1:1000 and 1:900), Moe increased approximately 10-fold during the first five days of the experiment and then dropped back to starting abundances. Incubations at an intermediate proportion of BQ3/Moe (1:500) resulted in an intermediate increase in Moe’s abundance starting at approximately 10^7^ gene copies mL^−1^ and increasing 100-fold to a final abundance of 10^9^ gene copies mL^−1^. These results demonstrated that Moe production saturated when incubations were started with high levels of Moe, and that some decay or loss of Moe influenced its final abundances in the 20-day batch cultures. Most importantly, though, these experiments provide clear evidence that Moe replicates in the presence of the putative Mesomimivirus BQ3.

Experiments were then conducted to determine if Moe was able to reproduce using the putative Phycodnavirus BQ1 [27] as its helper virus. In mixed infections with BQ1 and Moe, *C. parva* cultures lysed, albeit more slowly than cultures infected with BQ3 alone (Figure 6A). In mixed infections with BQ1 and Moe, Moe abundances dropped, whereas BQ1 gene abundances increased dramatically (Figure 6B), demonstrating that Moe does not replicate in the presence of infectious BQ1. This observation is consistent with knowledge of other isolated virophages and PLVs, which all replicate with a *Nucleocytoviricota* helper virus in the order *Imitervirales* [11,44], and while there is evidence of virophages associated with phycodnaviruses [6], these associations are based on metagenomics and have not been experimentally demonstrated.

Confirming the hyperparasitic replication strategy of Moe raised questions about its effects on its helper viruses and host cells. Mixed-infection experiments demonstrated that Moe inhibits BQ3 replication in a density-dependent manner. Every experimental trial included infections with BQ3 alone, and these single-virus infections resulted in rapid lysis of host cells (Figure 3, Supplementary Figure S3) even at the low MOI used (0.01 infectious units per cell). In mixed infections, BQ3 replication was observed; however, the extent of replication was modulated by the relative abundance of Moe. At lower Moe-to-BQ3 ratios, Moe exerted a comparatively small but statistically significant inhibitory effect on BQ3 replication, and as the relative abundance of Moe/BQ3 was increased to 500:1 and higher, BQ3 replication was suppressed; day-9 BQ3 abundances were ~300-fold lower in the 1000: 1 mixed infection compared to corresponding infections with BQ3 alone (Figure 5). Observations of reduced BQ3 replication in mixed infections with Moe were also associated with increased survival of *C. parva*, with some cultures maintaining relatively high cell abundances over the course of the 20-day incubations. For example, in the mixed infections with Moe/BQ3 inoculated at 1000:1 and 900:1, *C. parva* abundances dropped slightly but remained above 80% of the abundances observed in the corresponding no-infection control cultures. At lower proportions of Moe in mixed infections (i.e., at 10:1 and 200:1 Moe/BQ3), protection of *C. parva* growth was less apparent, and lysis patterns resembled the BQ3-only infections. Collectively, these results indicate a negative relationship between Moe density and BQ3 replication, and a positive relationship with *C. parva* growth. This was a surprising result considering that Moe’s closest cultivated relative, Gezel-14T, did not rescue its cellular host from lysis [26]. Although some virophages, like Mavirus and Sputnik, provide host protection, others, like Zamilon, do not [9], highlighting the diversity of interactions in tripartite infections involving virophages and PLVs.

Interestingly, *C. parva* seemed to recover at the end of the incubations with mixed infections at the lowest proportions of Moe. In these infections, *C. parva* abundances returned to 20% of the abundance in the control flasks by the end of the incubation (Figure S3B). Some recovery of *C. parva* was even observed in the BQ3-only infections, suggesting the existence of a small population of cells resistant to BQ3 infections (Supplementary Figure S3). Because the experiments described here were based on batch cultures, cell abundances did not continue to rise after this time point, and as consistently observed for *C. parva* cultures, all cells disappeared within 28 days of the initial inoculum, even for control cultures that were not infected. This pattern of growth in some infections suggests that infected *C. parva* cultures may be able to recover via a resistance mechanism that has not yet been studied. This phenomenon will be interesting to follow up on in future work on this system.

The precise mechanism through which Moe inhibits BQ3 replication is unclear. Moe may interfere with one or multiple stages of the giant virus replication cycle. Studies of other virophage systems have noted disruption of giant virus morphogenesis resulting in the production of defective particles, or a general inhibition of giant virus particle production by hijacking replication machinery or delaying the appearance of virus factories [11]. Additional studies will be required to determine the mechanisms of Moe’s influence on the replication of its helper viruses and growth of its host cells. It is also worth noting here that there is currently no evidence that Moe can exist as a provirophage element in its helper viruses or host cells, as observed for other virophage systems, e.g., [48,49,50], including Moe’s close relative, Gezel-14T [26]. Throughout the experiments described here, qPCR of purified *C. parva* genomic DNA or BQ3 lysate DNA with Moe primers and probes has never produced detectable amplification. Like the diverse replication strategies of virophages and related satellite viruses, the ability of virophages to integrate into host genomes does not appear to be an evolutionarily conserved aspect of these hyperparasitic viruses. Nonetheless, tripartite infection systems like *C. parva*-BQ3-Moe highlight the fascinating biology of cellular parasites. The fact that Moe appears to exert a density-dependent influence on its helper virus’s replication and host cell survival underscores the complex ecology of these systems and raises even more questions about algal virus evolution and persistence, and the diversity and ecology of viral hyperparasites.

## 5. Conclusions

A new virus that infects *C. parva*, CpV-BQ3, was discovered through our efforts to purify individual viruses of *C. parva*. Sequence analysis of BQ3 demonstrated its close relationship with *Tethysvirus ontarioense* (BQ2), yet it remains unclear if BQ3 should be considered a strain of *T. ontarioense* or a new species. Further, CpV-PLV Moe was co-isolated during the isolation of BQ3. TEM imaging of purified Moe particles revealed their morphological similarity to other virophages like Sputnik and mavirus [44] and PLVs like Gezel-14T [26]. Mixed infection experiments demonstrated that the putative Mesomimivirus BQ3 supports the replication of Moe, whereas the putative Phycodnavirus BQ1 does not. In these experiments, Moe influenced both BQ3’s replication and the survival of *C. parva* cells in a density-dependent manner, whereby high initial proportions of Moe relative to BQ3 hindered BQ3’s replication and mitigated *C. parva* lysis. Together with studies of other isolated virophages, the results reported here demonstrate the diverse replication strategies and impacts of hyperparasitism among members of the *Polisuviricotina*, the subphylum of the Preplasmiviricota that includes virophages and Polinton-like viruses [18].

## Figures and Tables

**Table 1:** Oligonucleotides used for qPCR (shown in 5′ to 3′ orientation).

Gene Target	Forward Primer	Reverse Primer	Probe *
BQ2 polB	AACGGATGACTTTATGAAA	CCCATTTGACCATATAATGA	TGTGCGATCATCTGCCTACCG
BQ3 polB	TGGGAGCAATAACTAGTG	CCATCAGAAACTTTAATAGCTA	ATGTAGCTGCTTCAACAACCGC
Larry MCP	CCAGGTACTACTTCCTTC	GAGACAAGTTGTTCAATGTA	TCTATTCCTCCACTTACAACCACCG
Curly MCP	TCGACCAGTACAAGAATA	GCTGAGTAGTTAGATCCA	TATTGTGTCCAGTGCTACCGCT
Moe MCP	GATCCTATTCAATGCGAAA	TGAGCGTGTATCATTAGG	TTCCAGCACCAGCCGATTCT

* All probes used 6-FAM as the 5′ dye and ZEN-3′ Iowa Black™ FQ as the 3′ quencher (IDT DNA).

**Table 2:** Cross-reactivity gene-specific qPCR primers and probes *.

		Gene Targets (5.0 × 10^9^ Gene Copies mL^−1^)					
		**BQ1**	**BQ2**	**BQ3**	**Larry**	**Curly**	**Moe**
Primers and Probe	BQ1	**14.9**	32.5	no Cq	35.0	33.5	25.0
	BQ2	no Cq	**14.8**	no Cq	35.0	32.0	24.0
	BQ3	no Cq	27.0	**14.1**	no Cq	no Cq	36.8
	Larry	36.7	no Cq	no Cq	**16.0**	30.7	23.7
	Curly	no Cq	no Cq	no Cq	33.3	**15.0**	20.3
	Moe	no Cq	no Cq	no Cq	No Cq	36.0	**17.2**

* Quantification (Cq) values are shown for each primer and probe set when amplifying all target molecules; “no Cq” indicates that amplification was not observed, and bolded values highlight amplification values for intended targets.

## Data Availability

The raw data supporting the conclusions of this article will be made available by the authors on request.

## Supplementary Materials

The following supporting information can be downloaded at https://www.mdpi.com/article/10.3390/v18020196/s1. Figure S1: Maximum Likelihood analysis of virus A32 genes; Figure S2: Maximum Likelihood analysis of virus VLTF-3 genes; Figure S3: Cell growth and virus replication for all experimental trials; Figure S4: Transmission electron microscopy of viral lysates.
