# Supplementary material for: A Polinton-like Virus of C. parva Inhibits the Population Growth of a Newly Isolated Relative of Tethysvirus ontarioense
Source: Viruses. 2026 Feb 1;18(2):196. doi: 10.3390/v18020196 (PMC12945077; doi:10.3390/v18020196)

Supplementary Figure Legends

Supplementary Figure S1. Maximum Likelihood analysis of virus A32 genes. The phylogeny was inferred using Maximum Likelihood and Jones-Taylor-Thornton model [1] of amino acid substitutions and the tree with the highest log likelihood (-10,757) is shown. The final dataset for the analytical procedure included 27 amino acid sequences with 345 positions.

Supplementary Figure S2. Maximum Likelihood analysis of virus VLTF-3 genes. The phylogeny was inferred using Maximum Likelihood and Jones-Taylor-Thornton model [1] of amino acid substitutions and the tree with the highest log likelihood (-15,024) is shown. The final dataset for the analytical procedure included 24 amino acid sequences with 509 positions.

Supplementary Figure S3. Cell growth and virus replication for all experimental trials. The data presented in columns are labelled at the top of the column; e.g., the first column of panels all present *C. parva* cell abundances. Each row of panels corresponds to a single experimental trial as described in the methods. Error bars represent the standard deviation of replicated experimental incubations.

Supplementary Figure S4. Transmission electron microscopy of viral lysates. (A - D) Micrographs obtained from samples of the 0.45 µm filtration lineage with highly abundant CpV-BQ3 as detected with qPCR. (E & F) Micrographs obtained from samples from the 0.20 µm filtration lineage within which both CpV-BQ3 and CpV-PLV Moe were detected with qPCR. The arrow in panel E points out a particle the same size as Moe.

Supplemental Figure S1

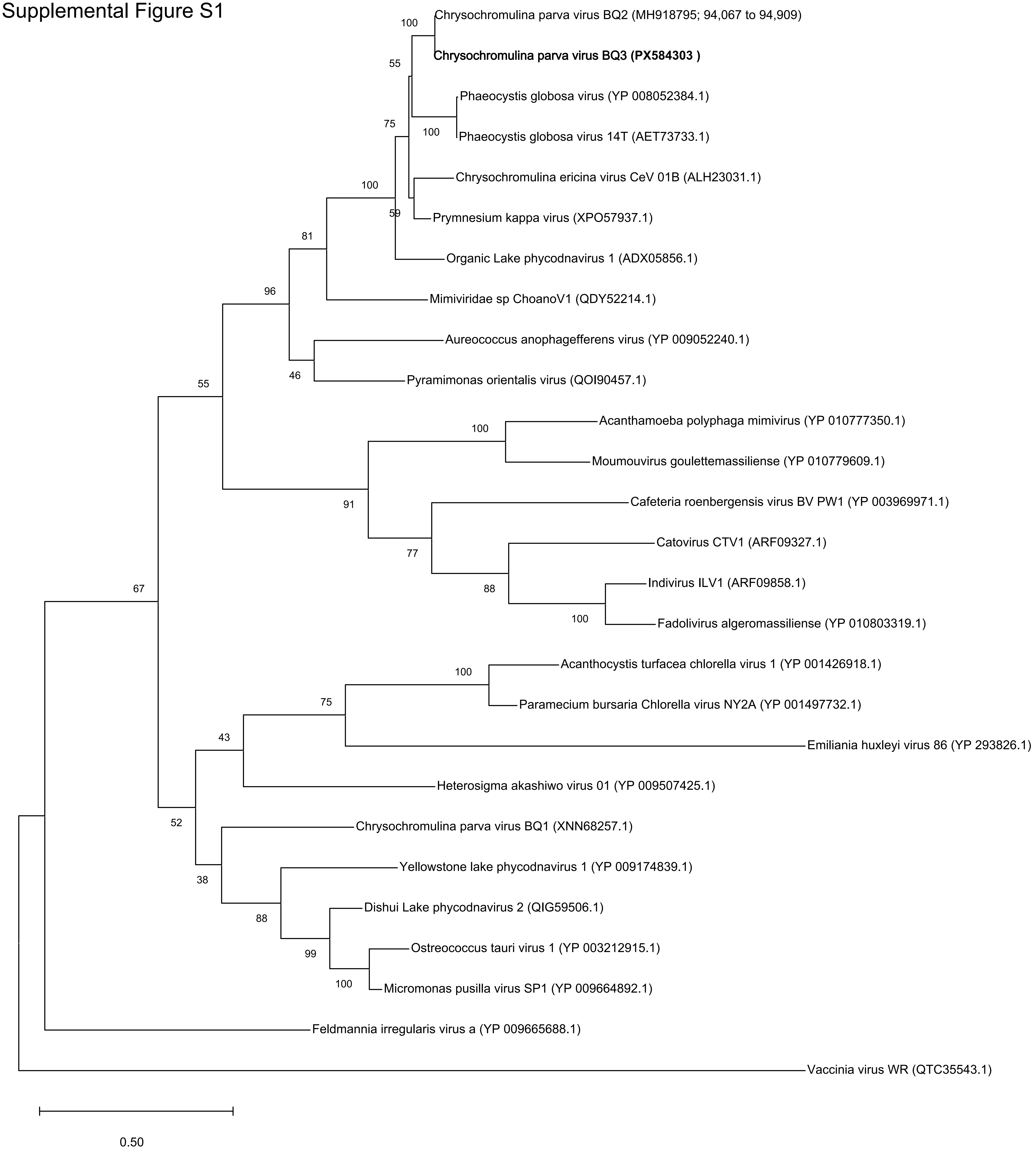

Supplemental Figure S2

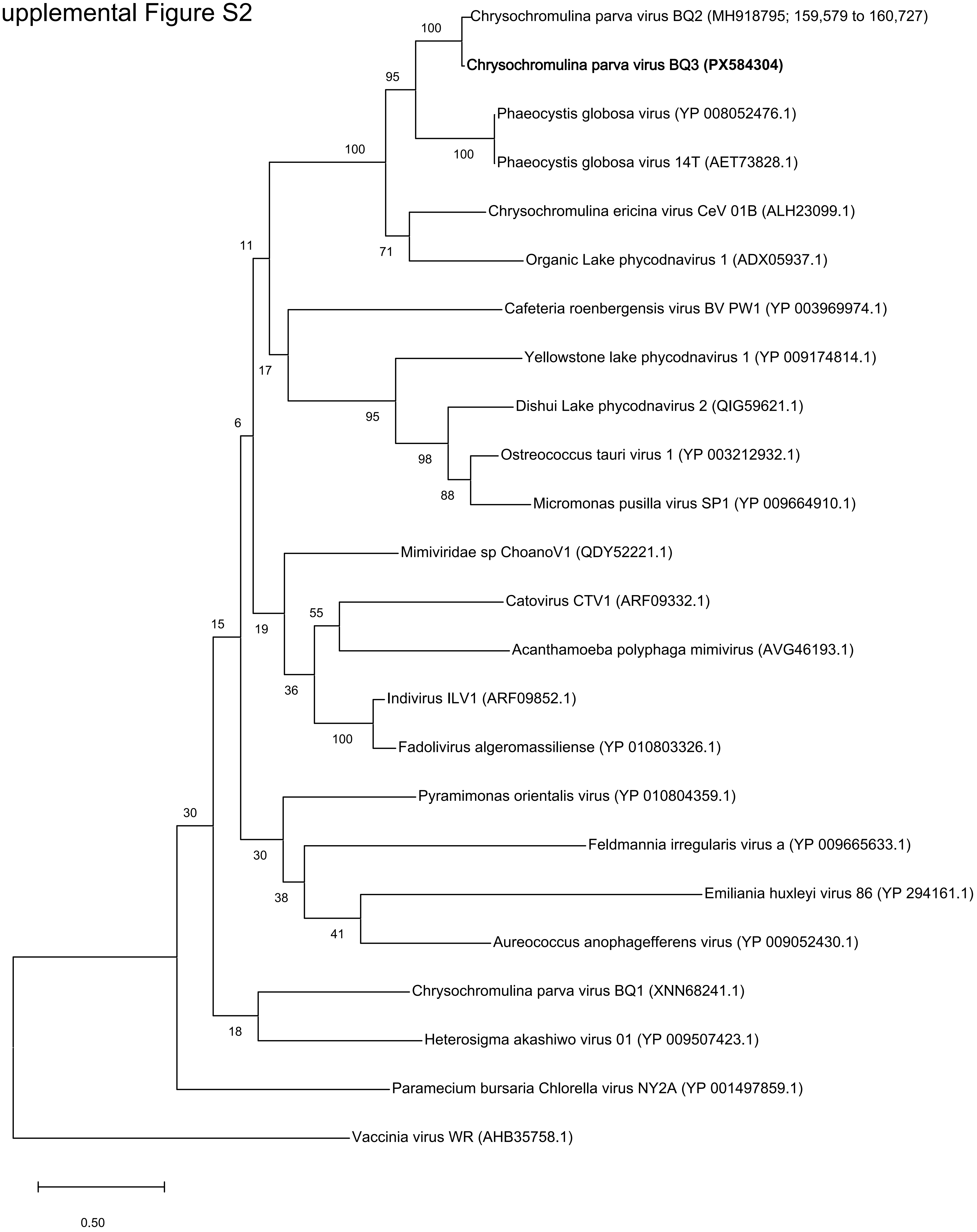

Supplemental Figure S3

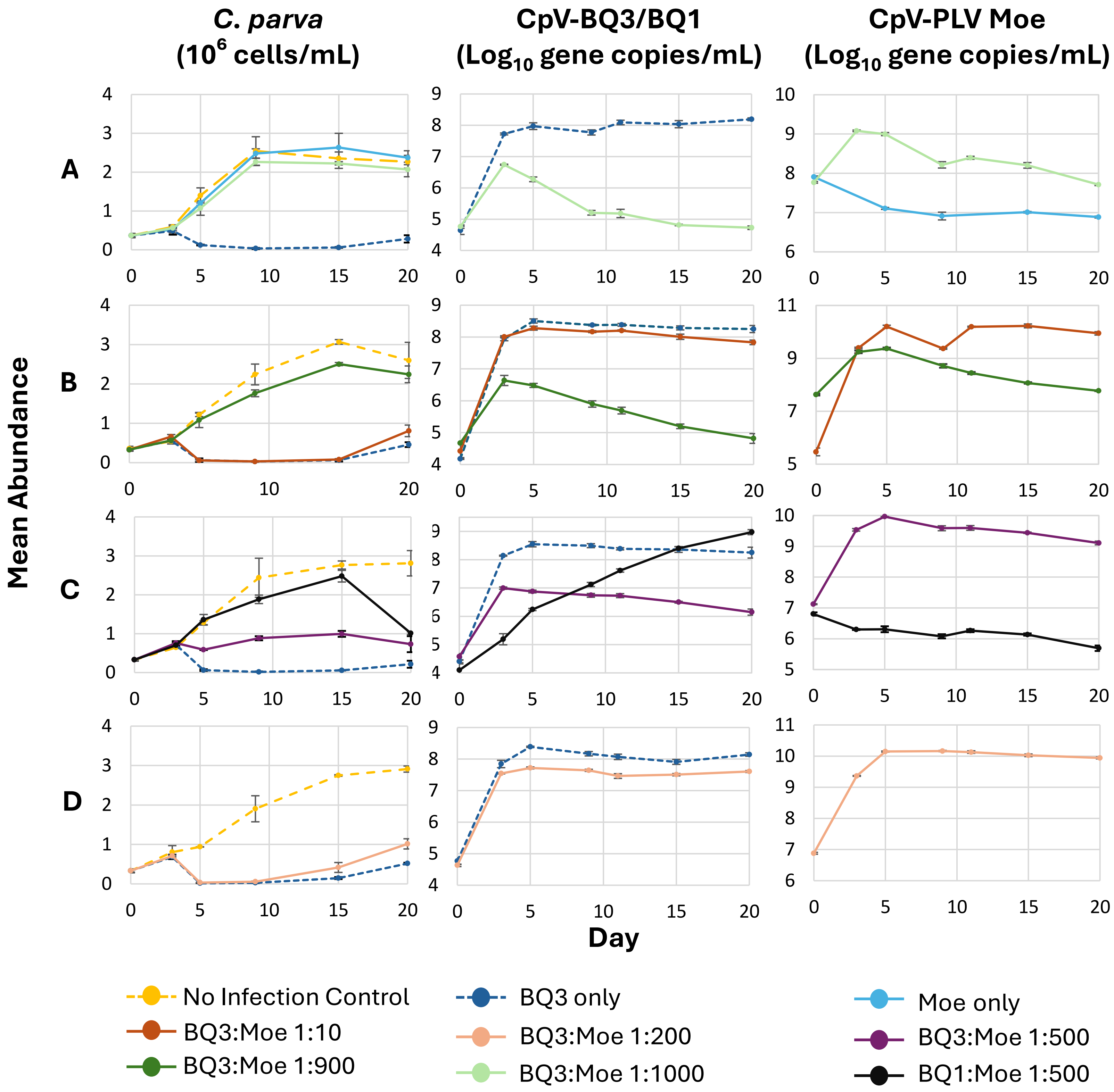

Supplemental Figure S4

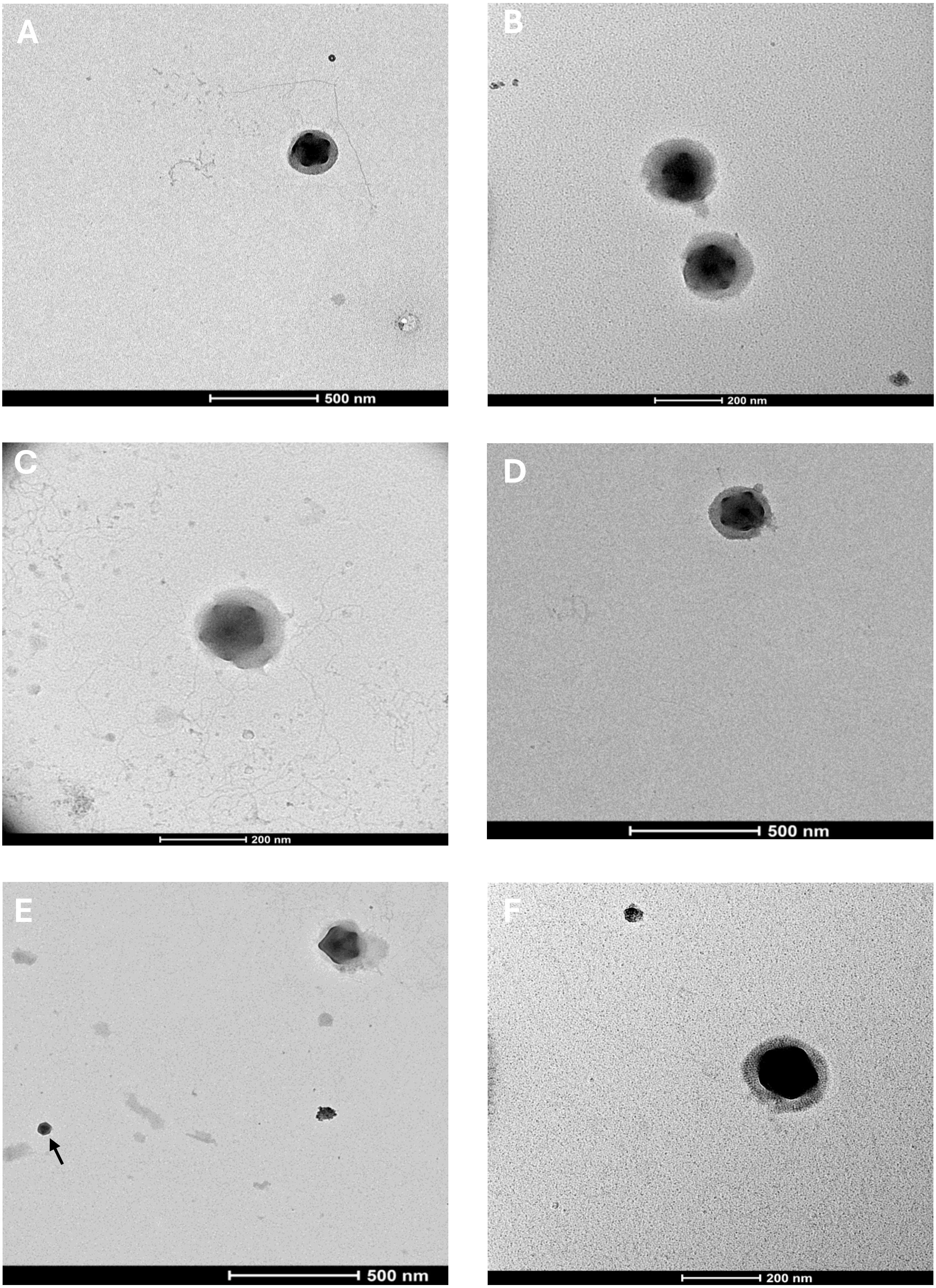

Supplement: Supplementary file 1 [file viruses-18-00196-s001.zip › viruses-4111968-supplementary.pdf]
